# Exploring human disease using the Rat Genome Database

**DOI:** 10.1242/dmm.026021

**Published:** 2016-10-01

**Authors:** Mary Shimoyama, Stanley J. F. Laulederkind, Jeff De Pons, Rajni Nigam, Jennifer R. Smith, Marek Tutaj, Victoria Petri, G. Thomas Hayman, Shur-Jen Wang, Omid Ghiasvand, Jyothi Thota, Melinda R. Dwinell

**Affiliations:** 1 Medical College of Wisconsin, Department of Surgery, Milwaukee, WI 53226, USA; 2 Medical College of Wisconsin, Department of Physiology, Milwaukee, WI 53226, USA

**Keywords:** Rat Genome Database, Genomics, Disease, Data analysis, Online resource

## Abstract

*Rattus norvegicus*, the laboratory rat, has been a crucial model for studies of the environmental and genetic factors associated with human diseases for over 150 years. It is the primary model organism for toxicology and pharmacology studies, and has features that make it the model of choice in many complex-disease studies. Since 1999, the Rat Genome Database (RGD; http://rgd.mcw.edu) has been the premier resource for genomic, genetic, phenotype and strain data for the laboratory rat. The primary role of RGD is to curate rat data and validate orthologous relationships with human and mouse genes, and make these data available for incorporation into other major databases such as NCBI, Ensembl and UniProt. RGD also provides official nomenclature for rat genes, quantitative trait loci, strains and genetic markers, as well as unique identifiers. The RGD team adds enormous value to these basic data elements through functional and disease annotations, the analysis and visual presentation of pathways, and the integration of phenotype measurement data for strains used as disease models. Because much of the rat research community focuses on understanding human diseases, RGD provides a number of datasets and software tools that allow users to easily explore and make disease-related connections among these datasets. RGD also provides comprehensive human and mouse data for comparative purposes, illustrating the value of the rat in translational research. This article introduces RGD and its suite of tools and datasets to researchers – within and beyond the rat community – who are particularly interested in leveraging rat-based insights to understand human diseases.

## Abstract (precis)

**Summary:** The Rat Genome Database (RGD) is the most comprehensive site for genetic, genomic and phenotype data for *Rattus norvegicus*, a leading animal model for disease-, drug- and toxicity-related research.

## Introduction

Since 1850, *Rattus norvegicus* (the laboratory rat) has been the model organism of choice for many investigations into the physiological mechanisms of complex diseases and the genetic and environmental factors that affect disease onset, progression and severity (Lindsey, 1979; Aitman et al., 2008). The more than 1.5 million publications of research using rat models reflect its use in laboratories around the world. Since the completion of the rat genome sequence in 2004 (Gibbs et al., 2004), more than 40 inbred rat strains commonly used as disease models have been sequenced and genomic variations among these strains identified, providing valuable tools for linking genotypes to phenotypes (Hermsen et al., 2015). Continued advancements in genetic-modification technologies have led to the generation of more refined models, further contributing to the increasing popularity of the rat as a genetic model of disease (Flister et al., 2015); the resulting targeted models provide important resources for researchers. Because of the recognized value of existing and emerging rat datasets, the Rat Genome Database (RGD; http://rgd.mcw.edu) was created in 1999 and has evolved into the leading resource for rat genomic, genetic, phenotype and strain data. The main responsibility of RGD is to retrieve rat data and confirm orthologous relationships with human and mouse genes. This collected and validated information is imported for use at several large data resources, such as Ensembl, NCBI and UniProtKB. RGD also provides official nomenclature for rat genes, quantitative trait loci (QTLs), strains and markers, as well as unique identifiers for each of these. In light of the current emphasis by publishers and funding agencies on the public availability of data and use of correct nomenclature and unique identifiers, rat researchers are increasing their data submissions to RGD for the appropriate assignment of symbols, names and stable identifiers, and for the public presentation of their research results. The value added by the RGD team through functional annotations of genetic and genomic elements, pathway analysis and visualization, and the integration of phenotype measurement data for strains used as disease models and control strains is immeasurable. By also providing comprehensive human and mouse data, further value is added, particularly to advance translational research. The interest and participation of the research community is reflected in the more than 670,000 RGD page views and the approximately 20,000 data file downloads (via FTP) made each year by individual researchers, university groups, research institutes and medical colleges, pharmaceutical companies, and bioinformatics and software developers. Here, we give an overview and update on RGD, with an emphasis on the tools and datasets related to the study of human diseases.

## Disease-related data acquisition

RGD provides complete gene, QTL and strain catalogues with comprehensive functional annotations for rat-, human- and mouse-derived data. Because many RGD users focus their studies on particular diseases, data are manually curated according to specific disease areas, providing an efficient means for curators to prioritize literature and integrate associated functional information. Genes, QTLs, strains and pathways related to the prioritized disease area are identified, the related literature is reviewed, and data are added to the database in the form of annotations to the appropriate ontologies (Table 1). Each annotation associates a data object such as a gene, QTL or strain with an ontology term and the reference that provides evidence for the association. RGD curators manually annotate disease and pathway information across species, gene ontologies for rat, and phenotype data for rat and human. These manually curated annotations are supplemented via software pipelines which, on a weekly basis, automatically import data from outside sources and associate those data with RGD genes as follows: Gene Ontology (GO) annotations for mouse and human genes are imported from the Gene Ontology Annotation (GOA) database (Huntley et al., 2015); Mammalian Phenotype Ontology (MP) annotations for mouse genes are imported from the Mouse Genome Database (MGD) (Bult et al., 2016), which is part of Mouse Genome Informatics (MGI), an international database resource for mouse research; human phenotype and disease annotations are imported through multiple pipelines: Online Mendelian Inheritance in Man (OMIM) (NCBI Resource Coordinators, 2016), ClinVar (Landrum et al., 2016) and the Genetic Association Database (GAD) (Becker et al., 2004), which has been retired, although data remain available; drug/chemical–gene interaction data are imported from the Comparative Toxicogenomics Database (CTD) (Davis et al., 2015); and data on molecular pathways are imported from the Kyoto Encyclopedia of Genes and Genomes (KEGG) (Kanehisa et al., 2016), the Small Molecule Pathway Database (SMPDB) (Jewison et al., 2014) and the Pathway Interaction Database (Schaefer et al., 2009) which, like GAD, is a retired legacy resource (Schaefer et al., 2009). As shown in Table 1, these data-acquisition efforts have resulted in enormous sets of annotations for diseases and disease-related phenotypes, as well as for functional categories such as pathways, biological processes and molecular functions, which can assist researchers in understanding how a gene or set of genes might be involved in the disease process. To provide easy access to these important datasets, RGD has created Disease Portals – entry points to consolidated disease-related data for researchers – and multiple software tools for data retrieval and analysis.

**Table 1.**
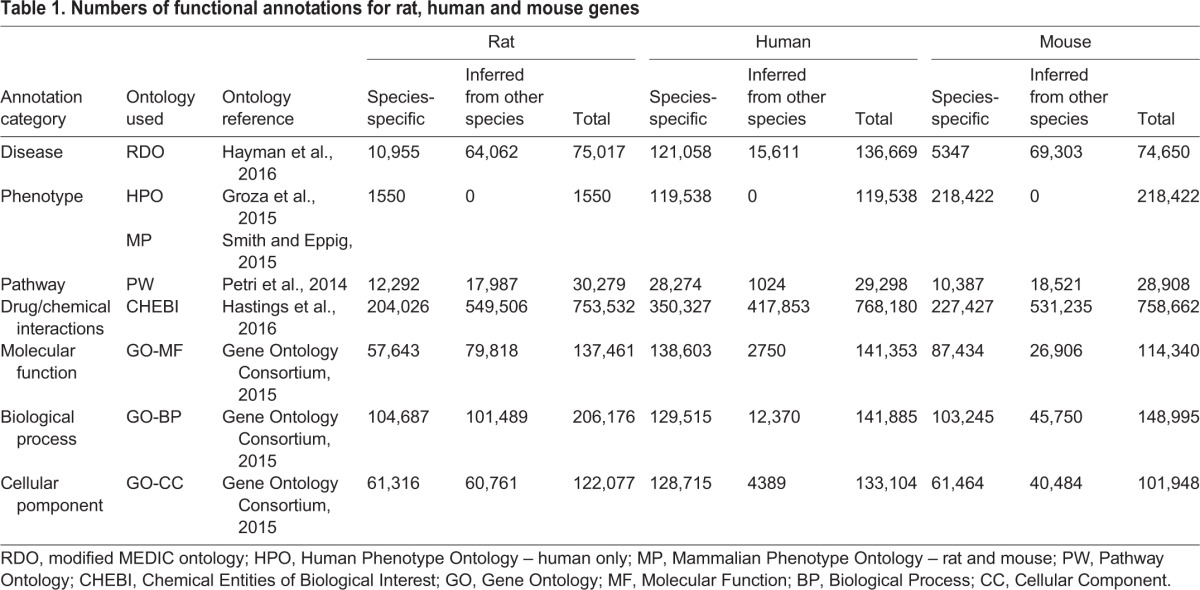
**Numbers of functional annotations for rat, human and mouse genes**

## Disease portals and data-mining tools

### RGD Disease Portals

**Fig. 1.**
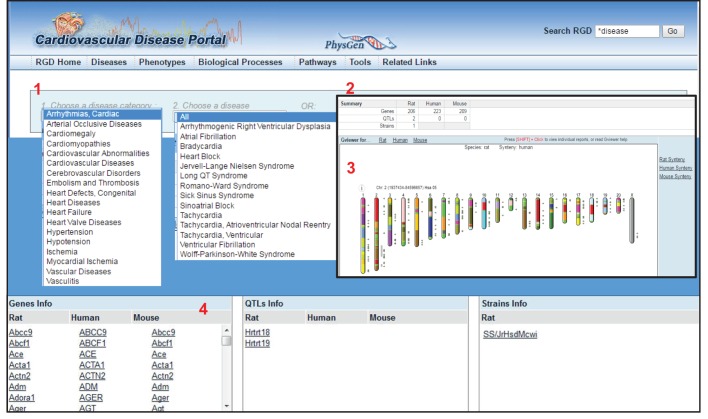
**The** **Cardiovascular Disease Portal home page.** Selecting ‘Arrhythmias, Cardiac’ in the first disease category dropdown menu (1) results in a summary view of rat, human and mouse gene, QTL and rat strain objects annotated to the selected term (2). Below that is a Genome Viewer (GViewer) display, showing the genomic positions of objects (genes, QTLs and strains) annotated to the term (3). These are presented in lists beneath the GViewer, with links to report pages dedicated to individual genes (4). Accessed 15 April, 2016.

**Fig. 2.**
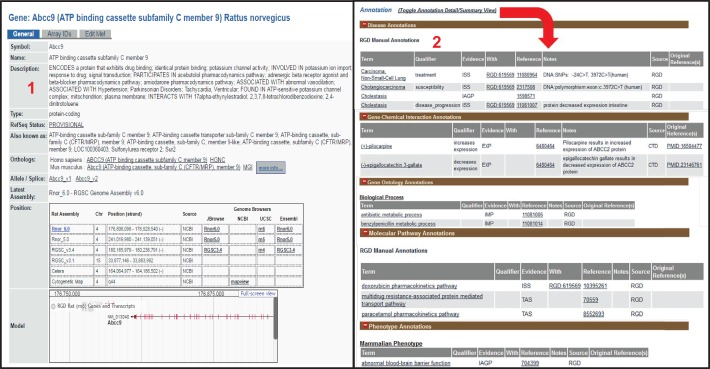
**A rat** **gene report page.** Each rat gene report page provides an annotation-based description, nomenclature, orthologs and mapping information for a specific gene, as well as other information (1). This is followed by expandable sections, which can be toggled to a more detailed display (red arrow), of different annotation categories for the gene – disease, gene–chemical interactions, GO, pathway and phenotype, with links to more detailed information about each annotation (2). Accessed 15 April, 2016.

Based on its manual-curation initiatives, RGD has generated Disease Portals, which provide easy access to multiple genomic and genetic data types associated with specific disease areas (Hayman et al., 2016). Currently, there are ten Disease Portals, covering: cardiovascular disease (CVD), cancer, diabetes, immune and inflammatory diseases, obesity and metabolic syndrome, and neurological, renal, respiratory, sensory organ and age-related diseases. Each portal integrates data for genes, QTLs and strains associated with the disease(s) highlighted by that portal. Each portal contains pages dedicated to different types of datasets, and these can be easily accessed via tabs at the top of each portal page. The different categories include ‘Diseases’, ‘Phenotypes’, ‘Biological Processes’ and ‘Pathways’. The dedicated pages each include a simple two-box search with dropdown menus. The first box contains major categories and, after a category is chosen, the second box presents the user with subcategories to choose from. For example, within the ‘Disease’ component of the cardiovascular Disease Portal, different types of CVD can be selected from the first dropdown menu, and further subcategories of the disease can be selected from the second dropdown menu (Fig. 1). The results shown in the main window include the number of genes, QTLs and strains associated with the selected disease term (Fig. 1, ‘2’), a visualization of them across the genome with a function to show human or mouse synteny (Fig. 1, ‘3’), and a listing of returned data elements annotated to the selected term for all three organisms with links to the respective report pages (Fig. 1, bottom). Report pages for genes, QTLs and strains provide summary views of annotations for diseases, phenotypes, drug/chemical–gene interactions, pathways and GO, with expanded views that include links to literature and other references from which the annotations were made (Fig. 2). Using the Disease Portals, researchers can get a fuller and better-rounded picture of their disease of interest, across three species.

### Ontology browser

**Fig. 3.**
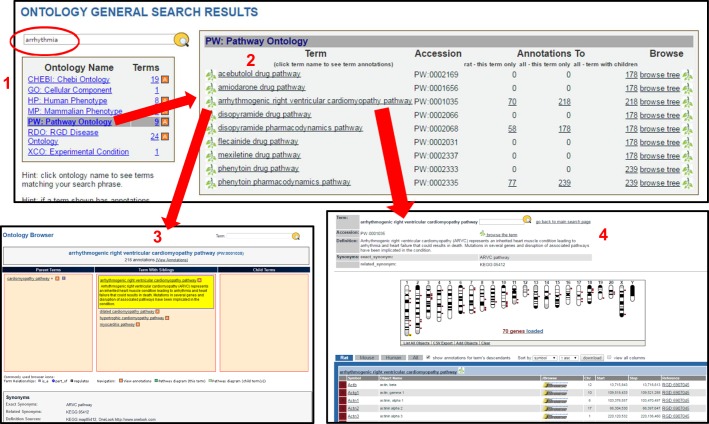
**Searching ontologies.** When searching for a term (circled), an initial report indicates which ontologies have terms containing the searched word (1). Clicking an ontology category provides a list of those terms (2). Clicking the branch icon next to a term places it highlighted in yellow in the Ontology Browser (3), with parent terms to the left, sibling terms below and any child terms to the right. Synonyms are provided at the bottom. Clicking on the term itself in the list (2) brings up the term ontology report page (4), which displays a Genome Viewer (GViewer) genome-wide view of objects annotated to the term. Below that is a list of rat, human and mouse genes annotated to the term, with links to the genome browser JBrowse to allow additional analysis. Accessed 15 April, 2016.

Another tool that allows easy access to multiple data types related to disease is the Ontology Browser, which can be accessed using the ‘Function’ button on the RGD homepage and with which a user can query across all the ontologies to find related data. Researchers can use a simple keyword search to retrieve the ontologies and respective terms associated with the search term (Fig. 3). Clicking on any of the ontologies will display the terms retrieved plus an indication of the number of existing annotations to that term and/or its more specific child terms (subcategories). In the example in Fig. 3, a search is made for the term “arrhythmia”. Seven ontologies had terms that contained this search text (Fig. 3, ‘1’); from these, we selected Pathway Ontology to view the matching terms in that ontology (Fig. 3, ‘2’). The user can then retrieve the data objects annotated to a specific term by clicking on that term in the table or on the corresponding annotation number. In Fig. 3, the term “arrhythmogenic right ventricular cardiomyopathy pathway” was selected: the ontology and its associated annotations can be explored further by clicking on the branch icon or the link labelled ‘browse tree’ (Fig. 3, ‘3’); clicking on the term itself links to an ontology report page that presents data elements annotated with the chosen term (Fig. 3, ‘4’). The page provides a karyotypic, genome-wide view of their locations, and a tabular list of the data elements (i.e. genes, QTLs and strains) with chromosomal locations and links to the JBrowse genome browser (for example, *Actb*, the first gene in the result list displayed in Fig. 3, links to a page that enables the user to view the individual elements more closely in their genomic context). The table also provides links to the corresponding gene, QTL or strain reports as well as the reference from which the annotation was made. Results include those for rat, human and mouse, and the data can be manipulated – for example by adding additional objects to or removing individual objects from the display – and/or downloaded using the functions available in the viewer.

### OLGA and the gene annotator

**Fig. 4.**
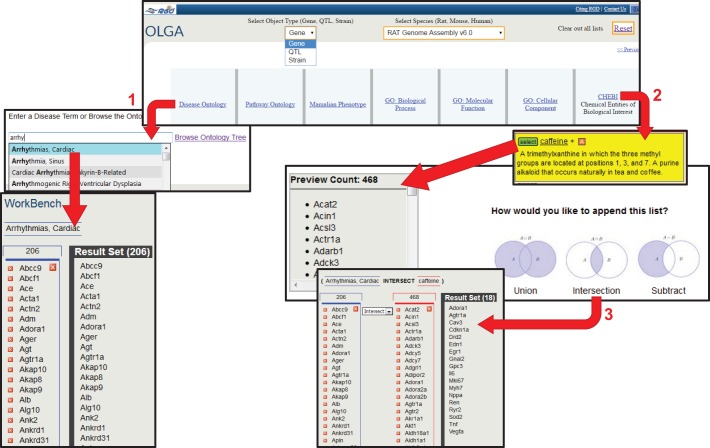
**The Object List Generator and Analyzer (OLGA) tool** **facilitates construction of complex queries for rat, mouse or human genes or QTLs, or rat strains.** In the example displayed, two queries are made for rat genes based on their functional annotations. In the first step (1), the Disease Ontology is searched. As the user types, an autocomplete list of disease terms is shown and the term “Arrhythmias, Cardiac” was selected. The resulting list, shown in the ‘WorkBench’ section of the page with the term used for the search, contains 206 genes. In the second step (2), the CHEBI ontology was browsed to locate the term “caffeine”. Selecting this term returns a preview list of 468 genes. Three options are given for appending the second list onto the first: ‘Union’, which combines the lists to produce the total non-redundant set of genes found in either list; ‘Intersection’, which returns the list of genes in common between the two; and ‘Subtract’, which returns only the genes from the first list that do not appear in the second. ‘Intersection’ was selected in this example (3) to produce a set of 18 genes that are associated with cardiac arrhythmias and interact with caffeine, with annotations. Accessed 15 April, 2016.

**Fig. 5.**
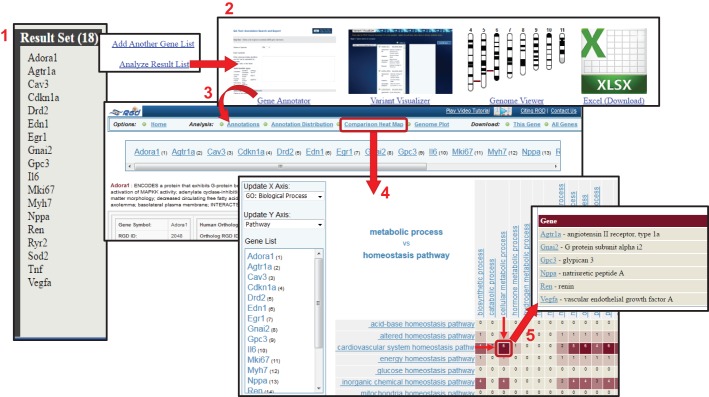
**The Gene Annotator (GA) tool** **displays detailed information about a list of genes and their functions, allowing commonalities in functional annotations between different genes to be explored.** The 18 rat genes (1) resulting from the OLGA query in Fig. 4 can be analyzed (2) in four different ways. From right to left, the list of genes can be downloaded as an Excel spreadsheet; viewed in the context of the entire rat genome using the Genome Viewer (GViewer) tool; used to search for strain-specific variants using the Variant Visualizer tool; or, as demonstrated here, explored using the Gene Annotator (GA) tool. Selecting the GA option (3) automatically populates the GA tool's search box with that list of genes. Alternatively, a user can access the tool from the ‘Genome Tools’ page of RGD and manually enter their gene list for analysis. The tool returns an individual report for each gene entered. The report displays the gene description, shows the orthologues of the gene, includes links to gene information hosted at other databases and lists the full set of ontology annotations for the gene and its orthologs. The Annotation Distribution function lists terms from seven functional categories and the percentage of input genes that share those annotations (not shown). The Comparison Heat Map function (4) allows users to view the number of genes at the intersection of two ontologies on multiple levels. Here, the user is comparing ‘GO Biological Process’ annotations with ‘Pathway’ annotations. By selecting terms along the horizontal and vertical axes, the user browses down the two ontologies to find the six genes that have annotations to the terms “cardiovascular system homeostasis pathway” and “cellular metabolic process” or to any more specific child terms in their respective vocabularies. Note that the colour intensity of the squares depends on the number of genes represented by each square, i.e. the more genes, the darker the colour. Clicking the dark brown square circled in red (5) opens a popup window showing the list of six genes at the intersection of these two ontological categories. Each gene symbol links to the RGD report page for more complete information about the gene. Because the original queries were for cardiac arrhythmias and caffeine, we know that these six genes have annotations for all four terms (and/or any more specific child terms under them): “Arrhythmias, Cardiac”, “caffeine”, “cardiovascular system homeostasis pathway” and “cellular metabolic process”. Accessed 15 April, 2016.

OLGA (Object List Generator and Analyzer) is a data-analysis tool that users can employ to assemble datasets for genes, QTLs or strains based on functional categories or genomic regions. Researchers can also upload gene lists and append or integrate these with gene lists created with the tool. Multiple lists of data objects can be created based on any of the categories. The lists can then be combined to create a non-redundant set, filtered to present just the common elements among the data sets, or subtracted, one list from another. Data mining for all three organisms (rat, mouse or human) is possible with OLGA. An example of dataset creation is presented in Fig. 4. The user initially chooses the data type (gene, QTL, strain) and then the method for creating the list [functional annotation (ontology), genomic position/region, QTL region, or symbol list upload]. In this example (Fig. 4, ‘1’), the user chooses to create a gene list based on the disease term ‘Arrhythmias, Cardiac’. The keyword search for each ontology offers an autocomplete feature and a list of potential matches so that the user can easily find the desired term. A list of genes annotated to ‘Arrhythmias, Cardiac’ or more specific categories of arrhythmias is assembled. At the time of access, the list contained 206 genes, but this number can vary as new annotations are added to the database. In step ‘2’, the user creates a second list of genes that interact with caffeine by browsing Chemical Entities of Biological Interest (CHEBI). This generated a list of 468 genes. The user then chooses how to integrate the two lists: through a union or intersection, or by subtracting the second list from the first (Fig. 4, ‘3’). In the example, an intersection of the two lists created a set of 18 genes that are associated with cardiac arrhythmias and also interact with caffeine. The user is then presented with the option to add another gene list or analyze the results (Fig. 5, ‘1’). ‘Analyze Result List’ presents the user with several options for displaying or analyzing the data aggregated to this point (Fig. 5, ‘2’). There is an option to download the gene set as a Microsoft Excel file, which includes the symbol, RGD ID, chromosome number and start/stop positions for each gene. The dataset can be displayed in the Genome Viewer, which shows the location of each of the genes beside the karyotype for that species, and also provides functions to add additional data objects or download the data. For rat genes, the gene list can also be uploaded into the Variant Visualizer tool in order to identify and visualize sequence variants within these genes across multiple strains. To obtain comprehensive data for each gene in the list and for further analysis of the dataset, the Gene Annotator (GA) tool is used (Fig. 5, ‘3’). Sending the gene set to the GA tool generates a full report for each gene, including human and mouse orthologue data. The report provides links to sequence data and corresponding gene reports at RGD, NCBI and Ensembl, as well as full functional annotations for GO, disease, phenotype, pathway and drug/chemical–gene interactions for all three organisms. Further investigation of the dataset can be conducted via the Annotation Distribution function, which shows the percentage of genes within the dataset associated with additional diseases, pathways, biological processes and other functional information. The Comparison Heat Map (Fig. 5, ‘4’) function provides an easy method for identifying subsets of genes based on their annotations. The user chooses functional categories for the *x* and *y* axes from the multiple ontology types in the provided dropdown lists. In this example (Fig. 5, ‘5’), the user chose ‘Pathways’ and ‘GO biological processes’ for the axes. Browsing down the ontologies by selecting terms on the axes restricts the results to genes annotated to terms in these more specific branches. In this way, the user narrows the original dataset of 18 genes to a subset of six genes that, from OLGA analysis, are associated with cardiac arrhythmias and interact with caffeine, and via the GA tool are shown to be involved in a cellular metabolic process and participate in a cardiovascular system homeostasis pathway. Links to the comprehensive gene reports are provided for this subset. As demonstrated, OLGA gives users the ability to perform complex queries for disease-related data and easily funnel those results into analysis tools for additional investigation.

## Summary

RGD provides a unique platform for accessing comprehensive disease-associated data for rat, mouse and human. Its Disease Portals centralize multiple data types related to specific disease areas within a single website component. Researchers can access gene, QTL, strain, pathway, phenotype and biological-process data that is of interest to them via a single resource. OLGA and the Gene Annotator are innovative, user-friendly software tools for creating and analyzing sets of genes, QTLs or strains related to multiple aspects of disease. RGD continues its commitment to providing the best in data and software tools for rat researchers and for researchers and clinicians beyond the rat research community.
